# The impact of one-decade ecological disturbance on genetic changes: a study on the brine shrimp *Artemia urmiana* from Urmia Lake, Iran

**DOI:** 10.7717/peerj.7190

**Published:** 2019-07-02

**Authors:** Alireza Asem, Amin Eimanifar, Gilbert van Stappen, Shi-Chun Sun

**Affiliations:** 1 Institute of Evolution and Marine Biodiversity, Ocean University of China, Qingdao, China; 2 College of Life Sciences and Ecology, Hainan Tropical Ocean University, Sanya, China; 3 Easton, MD, USA; 4 Laboratory of Aquaculture and Artemia Reference Center, Faculty of Bioscience Engineering, Ghent University, Gent, Belgium

**Keywords:** Climate change, Urmia lake, *Artemia*, Genetic variation, Historical mutations, Recent mutations, Demographic history

## Abstract

Urmia Lake, the largest natural habitat of the brine shrimp *Artemia urmiana*, has progressively desiccated over the last two decades, resulting in a loss of 80% of its surface area and producing thousands of hectares of arid salty land. This ecological crisis has seriously affected the lake’s native biodiversity. *Artemia urmiana* has lost more than 90% of its population during the decade from 1994 (rainy period) to 2004 (drought period) due to salinity increasing to saturation levels (∼300 g/l). We studied the influence of this ecological crisis on the genetic diversity of *A. urmiana* in Urmia Lake, based on one cyst collections in 1994 and 2004. AMOVA analysis on ISSR data demonstrated a 21% genetic variation and there was a 5.5% reduction of polymorphic loci between samples. PCoA showed that 77.42% and 68.75% of specimens clustered separately in 1994 and 2004, respectively. Our analyses of four marker genes revealed different genetic diversity patterns with a decrease of diversity at *ITS1* and an increase for *Na^+^/K^+^ ATPase*. There was no notable difference in genetic variation detected for *COI* and *16S* genes between the two periods. However, they represented distinctly different haplotypes. *ITS1* and *COI* followed a population expansion model, whereas *Na^+^/K^+^ ATPase* and *16S* were under demographic equilibrium without selective pressure in the 1994 samples. Neutrality tests confirmed the excess of rare historical and recent mutations present in *COI* and *ITS1* in both samples. It is evident that a short-term ecological disturbance has impacted the genetic diversity and structure of *A. urmiana*.

## Introduction

Urmia Lake (37°42′N, 45°19′E) is a landlocked thalassohaline lake with oligotrophic characteristics located in Northwest Iran. Its historical water surface area has ranged from 4,750 to 6,100 km^2^ with the average and greatest recorded depths being 6 and 16 m, respectively (Azari Takami, 1993; Van Stappen, Fayazi & Sorgeloos, 2001). It is among the largest hypersaline lakes in the world, like Great Salt Lake, USA, which has an average surface area from 4,400 to 8,500 km^2^ (Abatzopoulos et al., 2006; USGS, 2013), and it is inhabited by the brine shrimp *Artemia urmiana* Günther, 1899.

Contrary to widespread opinions, Urmia Lake and its adjacent wetlands are the habitat of various organisms. Based on its unique biodiversity, environmental gradients, socio-economic importance and existence of indigenous communities, Urmia Lake has been registered as a protected area since 1967 and as a national park since 1975. Because of its importance for migratory birds, it was also registered in the Ramsar Convention on Wetlands as a wetland of international importance in 1975 and considered as one of the 59 biosphere reserves by UNESCO in 1976 (Eimanifar & Mohebbi, 2007; Asem et al., 2014; Asem, Eimanifar & Sun, 2016; Asem, Eimanifar & Wink, 2016).

In recent years, many aquatic ecosystems have been subject to severe ecological changes. These alterations are imposing a considerable threat to local human societies in general (Biemans et al., 2011; Fernandes et al., 2011; Haddeland et al., 2014; Santos et al., 2014; Farokhnia, 2015). A progressive drought has increased the salinity of Urmia Lake from 170 g/l in 1994–1996 to more than 350 g/l (supersaturated) (Sorgeloos, 1997; Ahmadi, 2005; Ahmadi, 2007; Asem, Mohebbi & Ahmadi, 2012). The persistence of these conditions has caused the lake to lose 80% of its surface area (Aghakouchak et al., 2015). The desiccation of Urmia Lake is due to the interaction of reduced rainfall and consequent increased evaporation from the lake, human activity (uncontrolled construction of dams and overuse of surface water resources), and environmental mismanagement (Farajzadeh, Fakheri Fard & Lotfi, 2014; Fathian, Morid & Kahya, 2014; Merufinia, Aram & Esmaeili, 2014; Aghakouchak et al., 2015; Jalili, Hamidi & Ghanbari, 2016; Hamzekhani, Saghafian & Araghinejad, 2016; Shadkam et al., 2016).

Historical records document that Urmia Lake has grappled with drought crises over centuries to the extent that locals were able to walk across the lake (about 20 km) via a paved road (Morier, 1818; Curzon, 1892). Additionally, it was reported that due to the lack of freshwater and food, herbivorous animals, inhabiting the islands within the lake, deserted the islands by swimming and migrated into the surrounding mountains (Binder, 1887).

**Figure 1:**
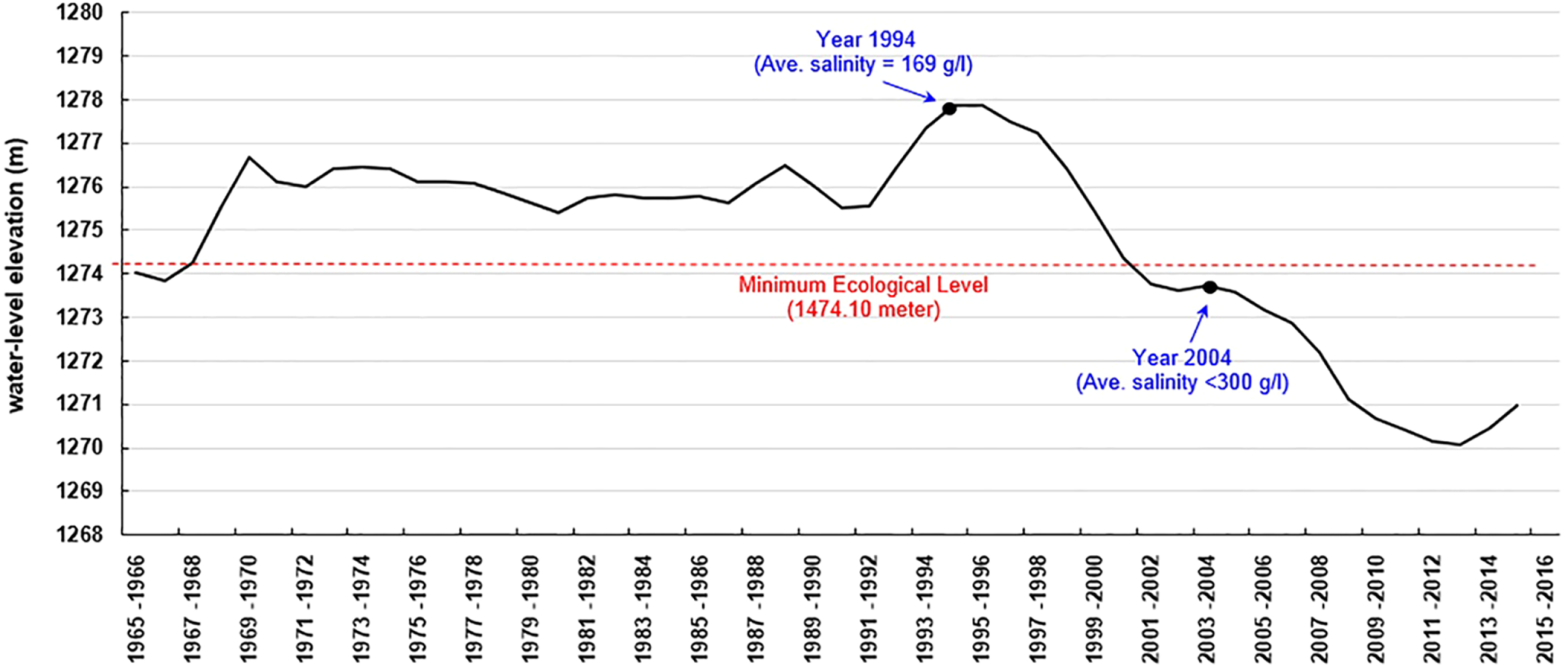
Annual average water level (elevation above the sea level) of Urmia Lake during 1965–2015 (AGRW, 2016).

Urmia Lake had the highest water-level elevation in 1994–1996 (1277.8 m a.s.l.) over the past six decades (from 1955 to 2015). Based on the first resource assessment of *Artemia* cysts and biomass in 1994–1995, *Artemia urmiana* cyst production in the upper 50 cm of the lake’s water column ranged from 4,200 to 4,500 tonnes/year (dry weight) (Sorgeloos, 1997). Analysis estimated that the cyst concentration was 399 cysts/l in that period (Asem, Mohebbi & Ahmadi, 2012). The water level of the lake fell below the “minimum ecological water level” after 2001 (1274.1 m a.s.l.; Abbaspour & Nazaridoust, 2007) (Fig. 1). Later estimates of *Artemia* cyst production declined to 27 and 25 cysts/l in 2003 and 2004, respectively, when salinity increased to saturated levels (∼300 g/l) (Ahmadi, 2005). In the following years cyst production dropped from an estimated 11 cysts/l in 2005 to 3 cysts/l in 2007 (Ahmadi, 2007). The lake lost most of its area after 2007 and no further assessments were performed, but some estimations indicated that the cyst concentration decreased to below 1 cyst/l (Asem, Mohebbi & Ahmadi, 2012). No live *Artemia* were observed in the main body of the lake during the summer of 2016, but did occur in the surrounding lagoons and estuaries (IARC, 2016). Now Urmia Lake has become an ecological disaster, receiving international attention. Iran’s Department of the Environment and the United Nations Development Programme (UNDP) ratified a project to save the lake and the surrounding wetlands (UNDP, 2014), at an estimated restoration cost of $1.3 billion (Hecht, 2014).

Critical environmental conditions can affect biodiversity and species distribution (Menendez et al., 2006; Barrett & Schluter, 2008; Jump, Marchant & Penuelas, 2009; Berkhout et al., 2014). Genetic diversity plays a decisive role in evolutionary history and future evolutionary directions of taxa (May, 1994; Forest et al., 2007; Jump, Marchant & Penuelas, 2009). While there have been many studies that focused on the effect of environmental changes on biodiversity, few studies have focused on intraspecific genetic variation (Pauls et al., 2013). Li et al. (1999), Li et al. (2000) and Li et al. (2001) documented significant genetic differentiation of wild emmer wheat (*Triticum dicoccoides*) in response to ecological change. A similar pattern was observed in slender oat (*Avena barbata*), followed by environmental variations (Jump, Marchant & Penuelas, 2009). A comparable pattern of genetic variation was observed in European white birch populations (*Betula pendula*), which showed different genotypes in warm and cool years (Kelly et al., 2003). An additional example was the observed significant negative correlation between *Gly-3* allele frequency and increasing summer precipitation in pinon pine (*Pinus edulis*) (Mitton & Duran, 2004).

Most studies on rapid evolutionary responses focus on morphological, physiological and nutritional variation. There are few studies that consider altered environmental conditions, especially short-term crises, on genetic variability (Thompson, 2009). Based on cyst collections and assessments in 1994 and 2004, *Artemia* has lost more than 90% of its reproductive potential and population size, and reproduction has stopped in the main body of Urmia Lake (Asem, Mohebbi & Ahmadi, 2012). We hypothesized that the tremendous changes in the environmental conditions of Urmia Lake and the reduction of the *Artemia* population size may have affected the genetic diversity. And, if the short-term ecological disturbance has influence on genetic variation, this change would be able to document in the intraspecific genomic dissimilarity. The specific objective of this study was to determine the independent impressionability of mitochondrial and nuclear genes through ecological crises.

## Materials and Methods

### Sampling strategy and DNA extraction

To assess intraspecific variation and population structure, we examined quiescent *Artemia* embryos collected in 1994 and in 2004 from Urmia Lake (Kholman-khaneh station; 45°29′E, 37°64′N). The samples were obtained from the upper 50 cm of the water column (see Sorgeloos, 1997). Because the bisexual *A. urmiana* coexists with a low ratio of a parthenogenetic population (Azari Takami, 1989; Agh et al., 2007) every specimen studied was first identified as belonging to the bisexual *A. urmiana* using SNP polymorphism in the *Na*^+^*/K*^+^ *ATPase α*-1 subunit (Manaffar et al., 2011), and re-certified by the phylogenetic analyses using the *COI* mitochondrial marker.

Total DNA of each specimen decapsulated embryo (number of specimens for each experiment below) were extracted following the Chelex® 100 Resin method (Bio-Rad Laboratories, Hercules, CA, USA). The embryos were crushed via a sterilized pipette tip, incubated for 2.5–3 h at 60 °C (tubes were shaken by vortex every 30 min) and eventually for 10 min at 80 °C. The tubes were centrifuged at 10,000 rpm for 1 min and the supernatant phase was directly used in the PCR reaction (Montero-Pau, Gómez & Muñoz, 2008; Eimanifar & Wink, 2013; Asem, Eimanifar & Sun, 2016; Asem, Eimanifar & Wink, 2016). The extracted DNA was stored at −80 °C for further genetic analyses.

### Genomic fingerprinting by ISSR-PCR

#### ISSR amplification

Nuclear genotype variation between *A. urmiana* samples collected in 1994 (31 specimens) and 2004 (32 specimens) was evaluated using inter-simple sequence repeats (ISSRs). ISSRs were amplified from genomic DNAs with two universal primers (GA)_8_T (Tulchinsky, Norenburg & Turbeville, 2012) and (AG)_8_YT (Eimanifar & Wink, 2013). PCR was carried out in a total volume of 20 µl containing 8 µl of ddH_2_O, 10 µl *Taq* polymerase (2 × TsingKe ™ Master Mix, Cat.# TSE004, TsingKe CO., CN), 1 µl template DNA and 1 µl of primer. The PCRs were carried out separately using the following conditions: 94 °C denaturation for 1 min, 35 cycles of 46–48 °C annealing for 50 s and 72 °C extension for 2 min. The final cycle was followed by a 7-min extension at 72 °C (Eimanifar & Wink, 2013). The final PCR products were visualized on 1.5% agarose gel (Cat.# 75510-019; Invitrogen, Carlsbad, CA, USA), run at 50 V for 3.5 h (for more information see Tulchinsky, Norenburg & Turbeville, 2012; Tiwari et al., 2015; Liew et al., 2015; Sharma et al., 2015).

#### ISSR statistics

The binary matrix (1 = presence; 0 = absence of a band) was determined for each year and population genetic information was computed separately. Genetic relationships among ISSR genotypes were established by principal coordinate analysis (PCoA) using GenAlex version 6.5 (Peakall & Smouse, 2012). The partition of genetic variation within and between 1994 and 2004 was determined using the Analysis of Molecular Variance implemented in GenAlex ver. 6.5 program (Peakall & Smouse, 2012).

### DNA sequencing

#### PCR amplification

Two fragments of nuclear markers (*Na*^+^*/K*^+^ *ATPase* and *ITS1*) and two mitochondrial markers (*COI* and *16S*) were amplified. PCRs were carried out in a total volume of 15 µl containing 6 µl of ddH_2_O, 7.5 µl Taq polymerase (2 × TsingKe ™ Master Mix, Cat.# TSE004; TSINGKE Biotechnology Co., Ltd., Chengdu, China), 0.3 µl template DNA and 0.6 µl of each primer. Sequencing was performed by TsingKe CO. (China).

A partial fragment of the nuclear gene, *Na*^+^*/K*^+^ *ATPase α-1 subunit*, was amplified using the primers of Manaffar et al. (2011). PCR amplification was performed under the following conditions: 94 °C for 2 min, 32 cycles of 94 °C for 25 s and 56 °C for 25 s and 72 °C for 1 min, and final extension with 72 °C for 3 min.

A fragment of the nuclear DNA containing a partial sequence of the 18S ribosomal RNA (*18S*), the complete sequence of internal transcribed spacer 1 (*ITS1*) and a partial sequence of the 5.8S ribosomal RNA (*5.8S*) genes, was PCR-amplified using the primers 18d-5′/R58 (Baxevanis, Kappas & Abatzopoulos, 2006). The thermal cycler PCR conditions were as follows: 4 min at 93 °C, 32 cycles of 40 s at 93 °C, 40 s at 62 °C, 1 min at 72 °C, and a final extension of 5 min at 72 °C.

Amplification of a partial fragment of the mitochondrial cytochrome oxidase subunit 1 (*COI*) gene was performed using the invertebrate universal primers L*COI* 490/HC02198 (Folmer et al., 1994). PCR amplification was carried out using the following program: a cycle of 3 min at 95 °C, followed by 35 cycles of one min at 95 °C, one min at 40 °C and one and half min at 72 °C, with a final step of 7 min at 72 °C.

The fragment of 16S ribosomal RNA (*16S*) was amplified using the primers *16S*-SP/*12S*-SP (Bossier et al., 2004). PCR amplification was carried out under the following conditions: 1 cycle of 94 °C for 2 min, 34 cycles of 1 min 15 s at 94 °C, 45 s at 52 °C, 2 min at 72 °C and a final extension cycle of 72 °C for 4 min.

Our DNA dataset consisted of 248 sequences including 70 specimens sampled for *Na*^+^*/K*^+^ *ATPase*, 60 specimens for *ITS1* and *COI*, 58 specimens for *16S* genes. The list of genetic markers and GenBank accession numbers is presented in Table 1.

**Table 1:** Population genetic indices for two *Artemia urmiana* samples collected in 1994 and 2004.

Markers	***Na***^***+***^***/K***^***+***^***ATPase***		***ITS1***		***COI***		***16S***	
Sampling year	**1994**	**2004**	**1994**	**2004**	**1994**	**2004**	**1994**	**2004**
N	35	35	30	30	30	30	28	30
GB	MK697598–MK697632	MK697633–MK697667	MK691705–MK691734	MK691735–MK691764	MK682320–MK682349	MK682350–MK682379	MK691599–MK691626	MK691627–MK691656
NS	198	198	1,150	1,150	647	647	875	875
S	1	4	93	59	41	38	62	67
Eta	1	4	96	65	41	39	64	70
H	2	4	29	24	24	22	28	29
Hd (±sd)	0.057 (±0.053)	0.166 (±0.084)	0.998 (±0.009)	0.963 (±0.027)	0.972 (±0.021)	0.961 (±0.023)	1.000 (±0.010)	0.998 (±0.009)
DHF	0.610^ns^ (±0.002)		0.475^ns^ (±0.021)		0.191^ns^ (±0.020)		0.760^ns^ (±0.013)	
*π* (±SD)	0.00029 (±0.0007)	0.00115 (±0.0839)	0.00751 (±0.0039)	0.00508 (±0.0027)	0.00525 (±0.0030)	0.00593 (±0.0009)	0.01041 (±0.0054)	0.01014 (±0.0053)
K	0.057	0.229	8.637	5.844	3.398	3.834	9.108	8.871
Exp. Het	0.057 (±0.000)	0.057 (±0.000)	0.092 (±0.070)	0.099 (±0.072)	0.083 (±0.037)	0.101 (±0.057)	0.146 (±0.126)	0.132 (±0.108)
Tajima’s *D*	−1.13^ns^	−1.88^*^	−2.45^**^	−2.42^**^	−2.47^**^	−2.24^**^	−1.70^ns^	−1.88^*^
Fu and Li’s D^*^	−1.732^ns^	−3.123^*^	−4.104^**^	−3.957^**^	−3.734^**^	−2.595^*^	−2.368^ns^	−2.534^*^
Fu’s *Fs*	−1.33^ns^	−3.12^ns^	−23.18^***^	−15.43^***^	−23.29^***^	−16.61^***^	−23.41^***^	−22.77^***^
BD	0.001		0.007		0.006		0.011	
*F*_ST_ (Pd)	0.000^ns^		0.011^ns^		0.001^ns^		0.016^ns^	

**Notes.**

N number of sequences GB GenBank accession numbers NS Total number of sites (excluding sites with gaps/missing data) S Number of polymorphic (segregating) sites Eta Total number of mutations H Number of haplotypes Hd Haplotype (gene) diversity DHF Differentiation of Haplotype Frequencies *π* Nucleotide diversity K Average number of nucleotide differences Exp. Het Expected heterozygosity BD Between group mean distance Pd Pairwise difference sd standard deviation

* *P* < 0.05

** *P* < 0.02

*** *P* < 0.001

ns non-significant (Fs should be regarded as significant if *P* < 0.02; Ashfaq et al., 2014)

### Sequence alignment and population genetic diversity

Sequences were aligned using MEGA ver. 6.00 with MUSCLE tool and default parameters (Tamura et al., 2013). Alignment lengths were 198, 1150, 665 and 875 bp for *Na*^+^*/K*^+^ *ATPase, ITS1*, *COI* and *16S,* respectively. Between-group mean distances (year 1994/2004) were computed using *p-distance* in MEGA ver. 6.00. To estimate the genealogical relationships among haplotypes for each gene, a maximum-parsimony haplotype network was inferred using the software TCS version 1.21 (Clement, Posada & Crandall, 2000).

For each marker, the number of polymorphic (segregating) sites (S), total number of mutations (M), number of haplotypes (H), haplotype (gene) diversity (Hd), differentiation of haplotype frequencies (DHF), nucleotide diversity (*π*), average number of nucleotide differences (K) and neutrality tests (i.e., Tajima *D*, Fu and Li’s *D* *, Fu’s *Fs*) were computed using DnaSP v.5.10 program (Librado & Rozas, 2009). Fixation index *F*_ST_(an overall population differentiation index) was calculated using Arlequin v.3.5 (Excoffier & Lischer, 2010).

## Results

### Species identification

The specimens analyzed in this study had a homozygous pattern (T-T) in the last valine codon using the *Na*^+^*/K*^+^ *ATPase α-1 subunit* with the exception of a single specimen collected in 1994 which showed a heterozygous pattern (T-G). Our phylogenetic trees (ML and BI) for *COI* showed that all analyzed specimens clustered with the reference sequence of *A. urmiana* (Maniatsi et al., 2011: HM998991) (Fig. S1). Only one specimen from 1994 placed in the diploid parthenogenetic clade also revealed a heterozygous pattern in valine codon; this sample was removed from the dataset.

### ISSR Profiling

**Figure 2:**
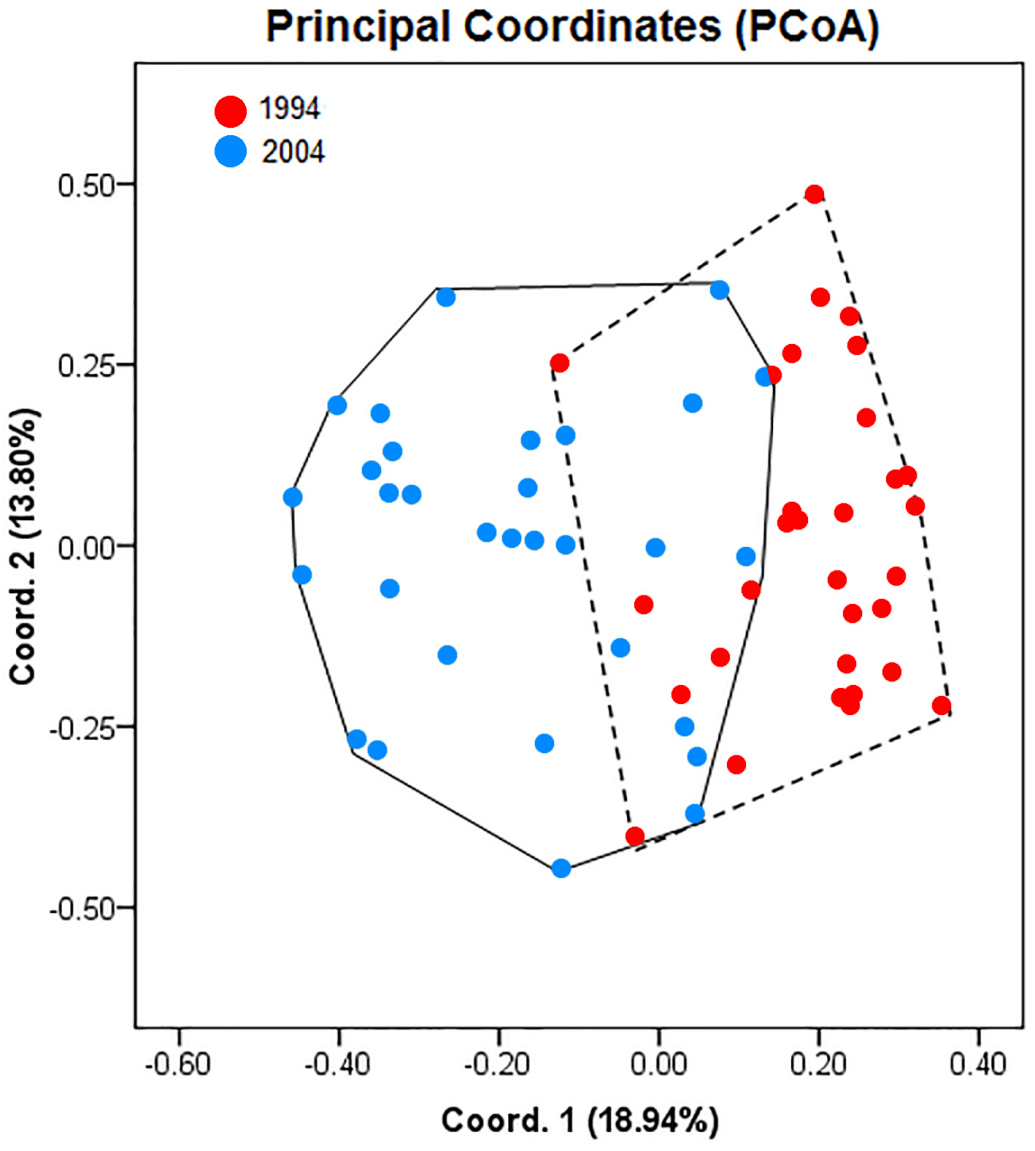
Principal coordinates analysis (PCoA) showing differentiation between *Artemia urmiana* samples collected in 1994 and 2004 using ISSR fingerprint genomic results.

A summary of population genetic indices of *A. urmiana* for all observed ISSRs loci between 1994 (rainy period) and 2004 (drought period) is listed in Table 2. ISSR profiling generated 17 bands with a single private band for each sample. The samples collected in 1994 and 2004 contributed 83.33% and 77.78% of polymorphic loci, respectively. AMOVA analysis demonstrated that 21% of genetic variation resided between the rainy and drought periods of *A. urmiana* (*df* = 1, SS = 20.116, *P*-value = 0.00001, Table 3). The first and second PCoA coordinates contained 18.94% and 13.80% of the variance, respectively (overall 32.74% of total variation). PCoA demonstrated that 1994 and 2004 were distinct groups, since there was only a narrow overlap between them (Fig. 2), with 77.42% and 68.75% specimens from the rainy and dry year being distinguished, respectively (Table 4).

**Table 2:** Summary of the genetic variation of all ISSRs loci observed for two *Artemia urmiana* samples collected in 1994 and 2004.

**Sample (year)**	**1994**	**2004**
Number of specimens	31	32
Number of bands	17	17
Number of private bands	1	1
Polymorphic loci (%)	83.33	77.78

**Table 3:** Molecular variation (within and among populations) for two *Artemia urmiana* samples collected in 1994 and 2004 (by AMOVA).

**Source**	**df**	**SS**	**MS**	**Est. Var.**	**Molecular variance (%)**
Among populations	1	20.116	20.116	0.572	21
Within population	61	128.837	2.112	2.112	79
Total	62	148.952	22.228	2.684	100

**Table 4:** PCoA results for two *Artemia urmiana* samples collected in 1994 and 2004, data shown as original count (percentage).

Year	Sample size	Unique area	Overlap
1994	31	24 (77.42)	7 (22.58)
2004	32	22 (68.75)	10 (31.25)

### Haplotype distribution

**Figure 3:**
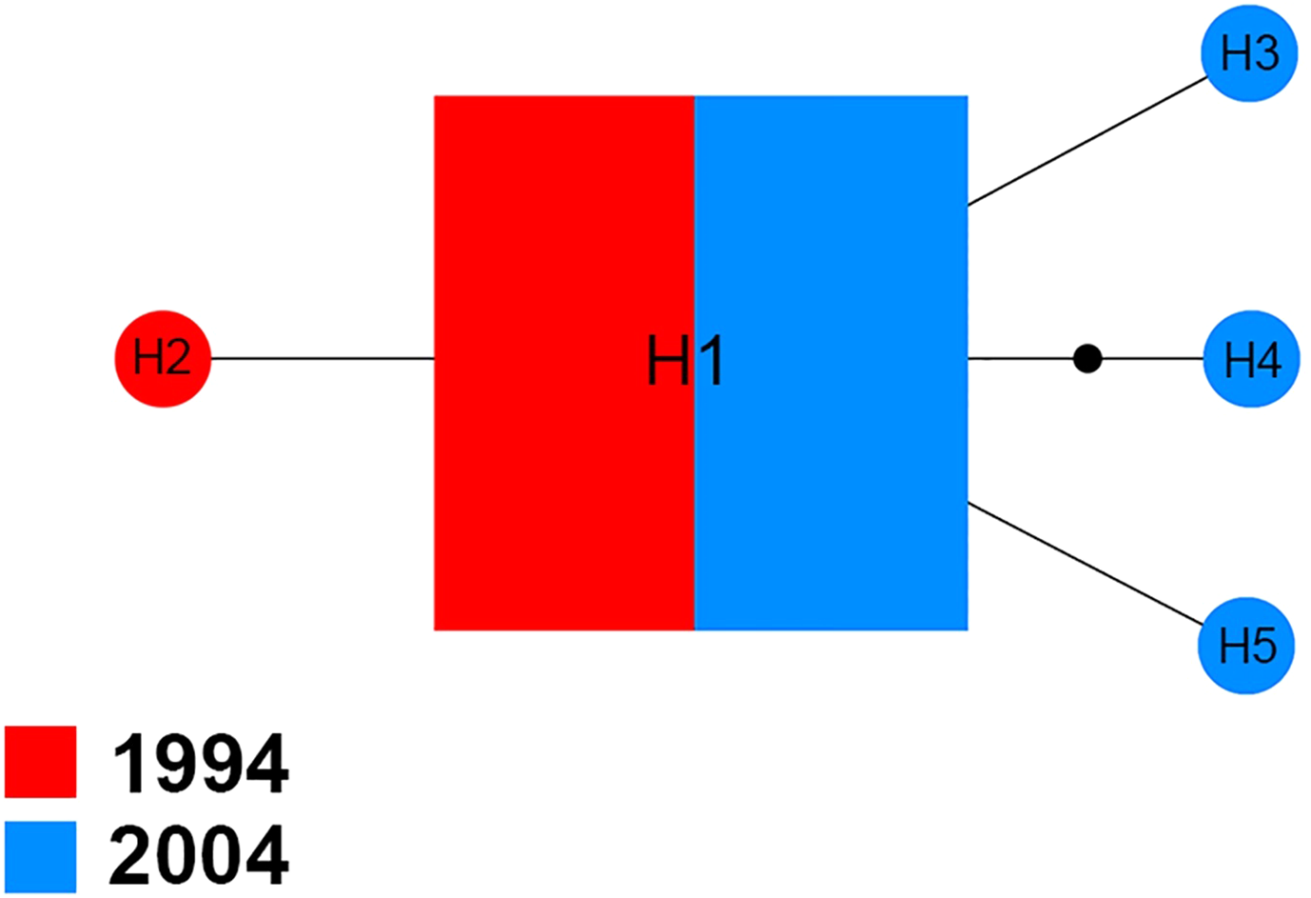
Maximum parsimony haplotype network of *Na^+/^K^+^ ATPase* sequences. The size of each square/circle is proportional to the frequency of specimens. Each joining line between haplotypes is equal with single nucleotide substitutions. B.

The *Na*^+^*/K*^+^ *ATPase* of 70 sequences produced five distinct haplotypes (H1–H5) for the 1994 and 2004 samples (Fig. 3). Among them, H1 was found in 94.3% (66/70) of specimens analyzed, including 51.5% (34/66) of specimens from 1994 and 48.5% (32/66) of specimens from 2004. In the four other haplotypes, one (H2) belonged to the 1994 sample and three (H3, H4 and H5) were only found in the 2004 sample.

**Figure 4:**
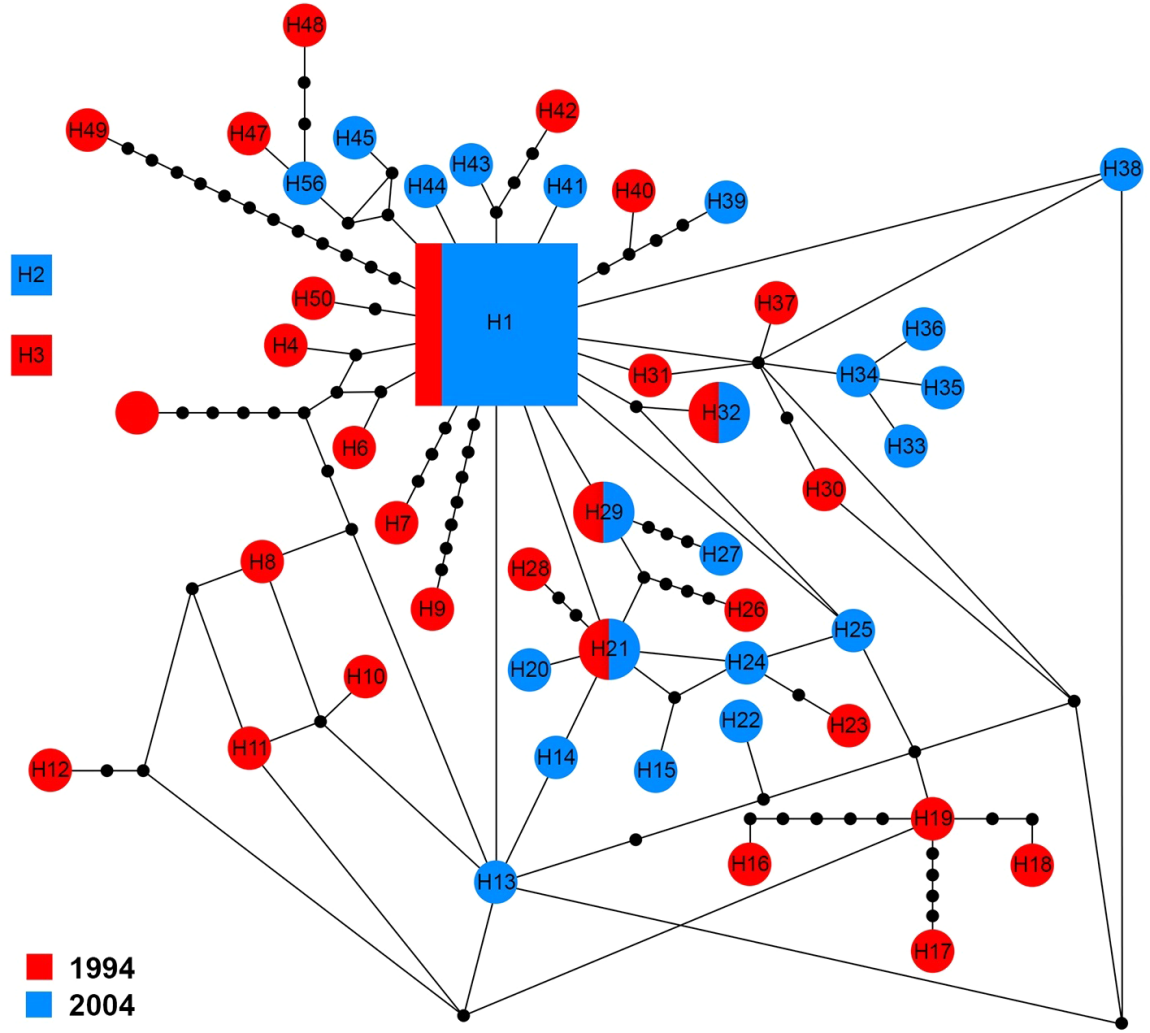
Maximum parsimony haplotype network of *ITS1* sequences. The size of each square/circle is proportional to the frequency of specimens. Each joining line between haplotypes is equal with single nucleotide substitutions. Black dots between haplotypes.

The *ITS1* sequences of 60 specimens showed 50 haplotypes (H1–H50). There were three groups of haplotypes (H1, H2 and H3) which were shared by 13.4% (8/60), 1.6% (1/60) and 1.6% (1/60) of specimens, respectively. H1 included two specimens from 1994 and six from 2004. With the exception of four haplotypes (H1, H21, H29 and H32) that shared genotypes between sampling years, other haplotypes were only found in one sampling year (Fig. 4).

**Figure 5:**
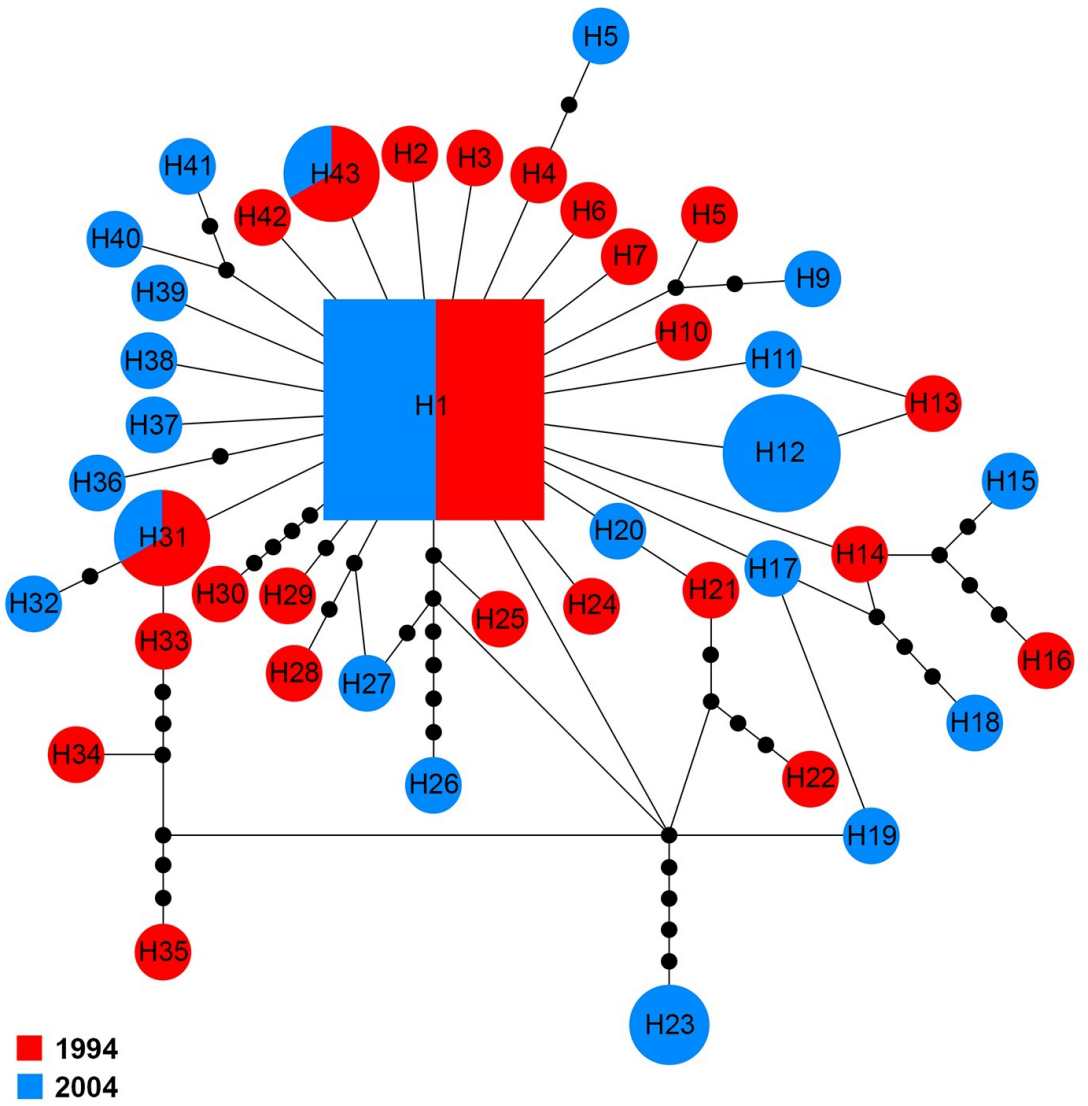
Maximum parsimony haplotype network of *COI* sequences. The size of each square/circle is proportional to the frequency of specimens. Each joining line between haplotypes is equal with single nucleotide substitutions. Black dots between haplotypes r.

The *COI* sequences for 60 specimens contained 43 haplotypes (H1–H43). Haplotype 1 was the major haplotype that was found in ten (16.7%) specimens. H12 and H23 were found in two and four specimens from 2004, respectively. H31 and H43 were shared by two specimens belonging to the two sampling years, while all other haplotypes came from a single sampling year (Fig. 5).

**Figure 6:**
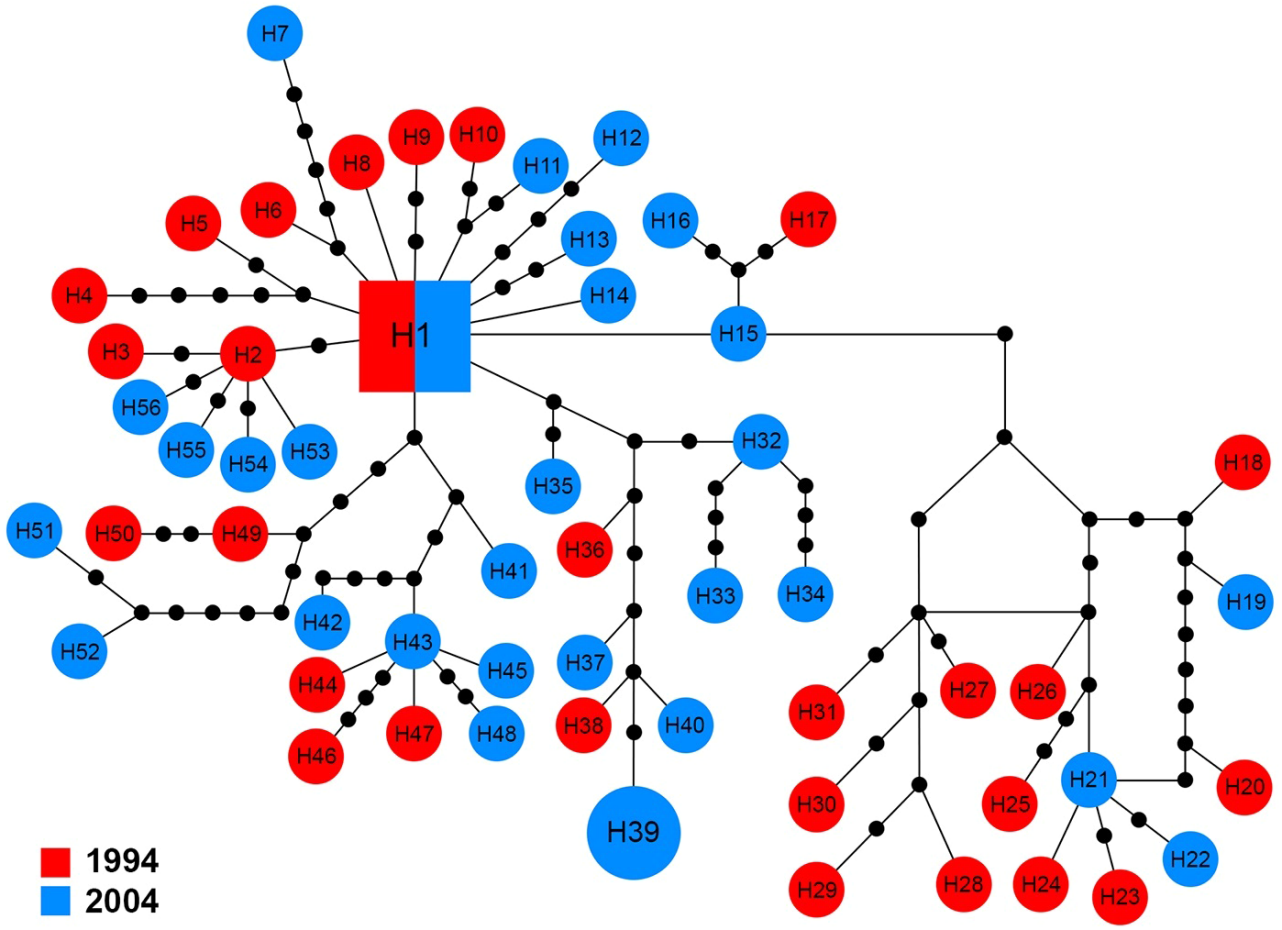
Maximum parsimony haplotype network of *16S* sequences. The size of each square/circle is proportional to the frequency of specimens. Each joining line between haplotypes is equal with single nucleotide substitutions. Black dots between haplotypes r.

The *16S* sequences for 58 specimens revealed 56 haplotypes (H1–H56), which showed high variation in comparison with the other markers. The central haplotype (H1) covered only two specimens (3.5%) including a single specimen from each year. With the exception of H39 that was shared by two specimens from 2004, other haplotypes were unique to a single specimen (Fig. 6).

### Genetic variation and neutrality tests

Genetic indices and allele frequency estimated for the aforementioned four markers are presented in Table 1. *Na*^+^*/K*^+^ *ATPase* showed the lowest population genetic indices in 1994. The haplotype diversity of *ITS1* had no remarkable difference between the 1994 and 2004 samples, but the other indices (*S*, *Eta*, *H*, *π* and *K*) demonstrated lower values in 2004 (drought period). The mitochondrial *COI* marker revealed a reduction of polymorphic sites, total number of mutations and number of haplotypes in 2004. In contrast, the number of polymorphic sites, total number of mutations and number of haplotypes of the *16S* marker showed lower values in 1994. Similar to *COI* there was no notable dissimilarity for the haplotype diversity, nucleotide diversity and average number of nucleotide differences in the *16S* marker between the two periods. Haplotype frequencies of all mitochondrial and nuclear markers represented non-significant difference between rainy and drought periods. Though there was no significant difference in the amount of genetic variation of *COI* and *16S* markers between rainy and drought periods, each marker presented different distinct of haplotypes. *16S* presented higher expected heterozygosity in 1994 while *ITS1* and *COI* showed higher values in 2004. The values of the pairwise genetic differentiation index (*F*_ST_) were not significant. The minimum and maximum between-group distances were detected in *Na*^+^*/K*^+^ *ATPase* (0.001) and *16S* (0.011), respectively. Neutrality tests yielded negative values with different significant and non-significant levels.

## Discussion

The effect of ecological disturbance, especially short-term regional climate changes, on genetic diversity is not well understood (Bálint et al., 2011; Banks et al., 2013). Ruediger et al. (2012) demonstrated that inter-annual and seasonal changes in water temperature produced significant variation in the genetic structure of *Daphnia* populations. The impact of salinity changes on genetic variation has been considered less frequently in aquatic organisms (Stoks, Geerts & De Meester, 2014).

Genetic variation and genetic diversity are important parameters to conserve biodiversity at all levels including population variabilities, individual fitness and adaptability of species to the environmental conditions (Amos et al., 2001; Hughes et al., 2008). Recently, Avolio, Beaulieu & Smith (2013) showed that the genotypic diversity of *Andropogon gerardii* (big bluestem grass) was significantly reduced after 10 years of an increase of experimentally-driven intra-annual precipitation variation. Brown et al. (2013) suggested habitat conflagration as a major critical process to reduce allelic richness of the mallee emu-wren *Stipiturus mallee* by reducing population size. Similar studies in zooplankton species such as *Artemia* have not been done. Environmental instability directly affects the *F*_ST_ (genetic differentiation among populations) through its impact on immigration and genetic drift combined with population reduction (Banks et al., 2013). Genetic diversity has been indicated to be important to population fitness since low levels of genetic variation may decrease the ability of population to adapt to the environmental crisis (Chapman et al., 2009; Pauls et al., 2013).

Although Urmia Lake is a wetland of international importance and is facing an acute ecological threat, few studies have assessed risks to its biodiversity. Asem et al. (2010) reported that in a rainy period (1994) the *Artemia* of this lake had a higher cysts size variation, significantly larger average egg size, and a thinner chorion than in a dry period (2004). The smaller cysts and thicker chorion produced during the dry period were attributed to decreasing food availability and to an acclimation mechanism, respectively, to increase the survivorship of the diapausing embryo under ecological crisis (Asem et al., 2010). Sankian, Heydari & Manaffar (2011) showed that *A. urmiana* hatching from cysts collected in 1998 (salinity = 180 g/l) had lower mortality but higher RNA content than those from 2003 (salinity approximately 300 g/l; saturated). Our ISSR fingerprint analysis on samples collected in 1994 and 2004 showed that each group had a single unique ISSR band. AMOVA analysis showed that 21% genetic variation occurred between the two periods; the drought period had lost 5.5% of polymorphic loci in comparison with the rainy period (Tables 2 and 3). Furthermore, the 1994 and 2004 collections were divided as two distinct groups, with 77.42% of the 1994 specimens and 68.75% of the 2004 specimens separated by PCoA. These results suggest that one decade of environmental changes has caused genetic structure and biometrical variation of cyst (see Asem et al., 2010) in this population.

Theoritically a decreasing population size in response to unfavorable ecological changes is expected to lead to a reduction of genetic diversity (Bálint et al., 2011; Cobben et al., 2011; Pauls et al., 2013). But in our study, the population genetic indices generated different patterns of genetic variation. Generally, results have demonstrated that the genetic variation of *ITS1* in the drought period was reduced when the salinity of the lake was increased near saturation (300 g/l). In contrast, the genetic diversity of *COI* and *16S* was not significantly different between the two periods. Additionally *Na*^+^*/K*^+^ *ATPase* revealed a remarkable increase in variation in the drought period. These conflicting results might be attributed to the difference in the potential of gene variability that might be confirmed by further experimental evidence.

Our study showed a negative and significant Tajima’s *D* value for both examined periods in *COI* and *ITS1* (Table 1), which indicated an excess of rare haplotypes resulting from population expansion or from selective sweeps (Nei & Kumar, 2000; Swanson, Aquadro & Vacquier, 2001; Akey et al., 2004; Cruciani et al., 2008; Levitan & Stapper, 2009). The negative values of Tajima’s *D* should be referred to the demographic expansion of these markers in both ecological periods with regard to developed haplotype networks of *ITS1* and *COI* markers. Additionally, Fu and Li’s D* and Fu’s *Fs* tests showed a negative departure from the neutrality test. Given that Fu and Li’s *D* * test and Fu’s *Fs* test recognize an excess of rare historical mutations (Fu & Li, 1993; Fu, 1996; Zhao et al., 2008), and rare recent mutations (Fu, 1997; Ramos-Onsins & Rozas, 2002; Zhao et al., 2008), respectively. Therefore the excess of both old and novel mutations were confirmed in the gene pool of *COI* and *ITS1* during the rainy and drought periods. Consequently, the results of neutrality tests of Fu and Li’s D* and Fu’s *Fs* could explain the patterns of haplotype networks and population expansion of *ITS1* and *COI*.

The major influence of short-term ecological disturbance was observed in the demographic history of *Na*^+^*/K*^+^ *ATPase* and *16S* markers. Neutrality tests resulted in a non-significant value for *Na*^+^*/K*^+^ *ATPase* in the rainy period, which supported the demographic equilibrium. While significantly negative Tajima’s *D*, Fu and Li’s D* strongly supported a demographic expansion and an excess of rare historical mutations in the drought period. These results are consistent with the pattern of haplotype distributions developed during this period. In addition, the non-significant value of Fu’s *Fs* suggested the absence of recent mutations in *Na*^+^*/K*^+^ *ATPase* in both periods.

Another major alteration was observed in the *16S* structure. Tajima’s *D* and Fu and Li’s D* were non-significant in the rainy period which could indicate *16S* marker was at demographic equilibrium without selection in the rainy period. In contrast, a negative and significant neutrality value and expanded haplotype network indicated that *16S* is involved in recent expansion in the drought period (Fig. 6). The negative and significant value of Fu *Fs* test suggested the excess of new mutations in the gene pool of *16S* in both normal and drought periods.

Overall, our results have demonstrated that ecological disturbance should be considered in hypotheses about effects of short-term environmental changes on genetic variation. The rapid genetic changes that we found has also been demonstrated in some other species of animals and plants experiencing environmental crises (Avolio, Beaulieu & Smith, 2013; Brown et al., 2013; Banks et al., 2013). This could be attributed to the hereditary potential of populations respond to immediate ecological changes (Thompson, 2009). Although previous studies have shown a decreasing genetic variation in response to ecological disturbance, we found that *Artemia urmiana* shows dissimilar responses to environmental changes. Consequently, changes in genetic diversity and the pathway of variation are controlled by interaction between ecological conditions and the ability of genes to vary. Rogers (2015) suggested phenotypic patterns can be affected by ecological conditions which may cause genetic variation within an anostracan population during different periods. It is evident that the ecological crisis at Urmia Lake has had a meaningful influence on *Artemia urmiana* genetic structure, especially reducing genetic diversity, which ultimately could risk the survival of this crustacean.

##  Supplemental Information

10.7717/peerj.7190/supp-1 Figure S1 *COI* phylogeny of *Artemia* based on BI and ML approach. The number behind major nodes denote posterior probabilities. *Daphnia tenebrosa* (HQ972028) was used as an outgroup

10.7717/peerj.7190/supp-2 Supplemental Information 1 Sequences of Na/K ATPas

10.7717/peerj.7190/supp-3 Supplemental Information 2 Sequences of ITS1

10.7717/peerj.7190/supp-4 Supplemental Information 3 Sequences of COI

10.7717/peerj.7190/supp-5 Supplemental Information 4 Sequences of 16S

## Additional Information and Declarations

### Data Availability

The following information was supplied regarding data availability: Na+/K+ ATPase data is available at GenBank: MK697598 to MK697667. ITS1 data is available at GenBank: MK691705 to MK691764. COI data is available at GenBank: MK682320 to MK682379. 16S data is also available at GenBank: MK691599 to MK691656.
