# Supplementary figures and images for: The impact of one-decade ecological disturbance on genetic changes: a study on the brine shrimp Artemia urmiana from Urmia Lake, Iran
Source: PeerJ. 2019 Jul 2;7:e7190. doi: 10.7717/peerj.7190 (PMC6611446; doi:10.7717/peerj.7190)

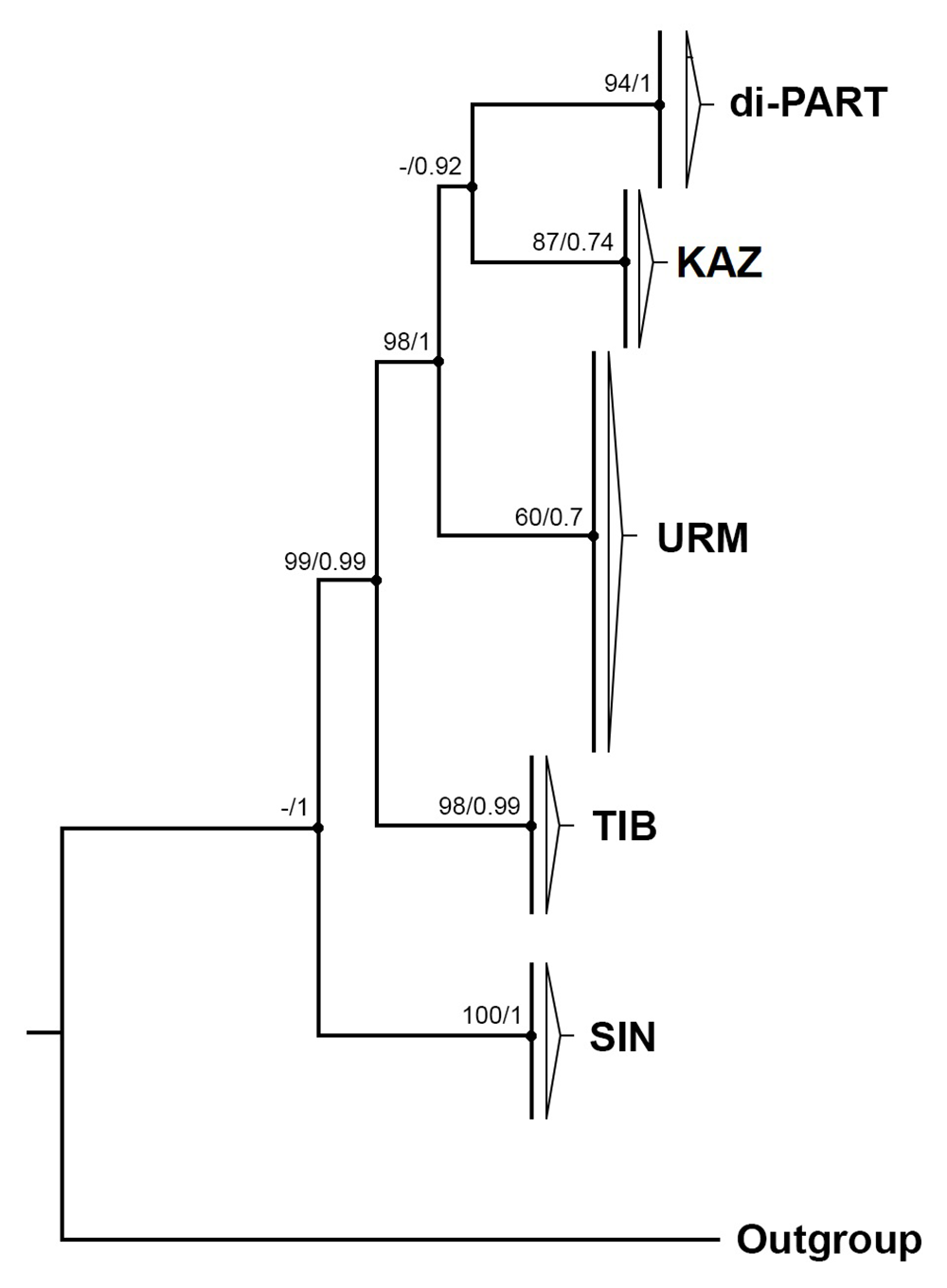

Supplement: Figure S1 [file peerj-07-7190-s001.png]
